# Protective Effects of Eicosapentaenoic Acid on the Glomerular Endothelium via Inhibition of EndMT in Diabetes

**DOI:** 10.1155/2021/2182225

**Published:** 2021-12-24

**Authors:** Toshinori Yasuzawa, Tomomi Nakamura, Shigeru Ueshima, Akira Mima

**Affiliations:** ^1^Department of Nephrology, Osaka Medical and Pharmaceutical University, Osaka, Japan; ^2^Department of Food Science and Nutrition, Faculty of Agriculture, Kindai University, Nara, Japan; ^3^Department of Health and Nutrition, Faculty of Health Science, Kio University, Nara, Japan

## Abstract

Diabetes-induced endothelial pathologies are hypothesized to lead to the progression of diabetic kidney disease (DKD). The endothelial to mesenchymal transition (EndMT) possibly induces fibrosis, leading to glomerulosclerosis in the kidney. Furthermore, this could lead to albuminuria in diabetic nephropathy due to glomerular endothelial dysfunction. Eicosapentaenoic acid (EPA), purified from fish oil, decreases inflammatory cytokine levels in glomerulonephritis. Here, we aimed at finding whether ethyl eicosapentaenoate (EPA-E) exerts renal protective effects via EndMT inhibition. To find out whether EPA inhibits EndMT *in vitro*, the changes in CD31 expression were studied in cultured mouse endothelial cells. The addition of the conditioned medium from the adipocyte culture significantly decreased the protein levels of CD31, while the addition of EPA-E partially reversed this inhibition. Further, EndMT inhibition by EPA-E treatment might occur via the inhibition of the protein kinase C*β* (PKC*β*)/transforming growth factor-*β* (TGF-*β*)/plasminogen activator inhibitor-1 (PAI-1) signaling and not via microRNAs. Streptozotocin-induced diabetic mice fed a high-fat diet (60% from fat) exhibited mesangial expansion and albuminuria. Induction of EPA-E ameliorated the mesangial expansion and decreased albuminuria without affecting blood pressure, triglyceride and free fatty acid levels, and intraperitoneal glucose. These findings suggest that EPA-E exerts renal protective effects on endothelial cells, by normalizing EndMT followed by the PKC*β*/TGF-*β*/PAI-1 signaling. Thus, EPA-E has the potential for imparting renal protection by regulating EndMT in DKD.

## 1. Introduction

The international diabetes foundation (IDF) reported that 463 million adults were diagnosed with diabetes in 2019, and approximately 700 million adults are expected to be diabetic by 2045 [1]. Diabetic kidney disease (DKD) is the most important chronic kidney disease (CKD), requiring renal replacement therapy [2]. Moreover, the prevalence of dialysis due to DKD-induced end-stage renal disease (ESRD) has exceeded that of glomerulonephritis-related ESRD in the U.S., and the number is increasing [3]. Several large clinical studies such as the action in diabetes and vascular disease: Preterax and Diamicron modified release controlled evaluation (ADVANCE) study, the UK prospective diabetes study (UKPDS), and the action to control cardiovascular risk in diabetes (ACCORD) trial indicated that intensive glycemic control could reduce the risk of DKD [4–7]. On the other hand, large randomized controlled studies including the ADVANCE study, ACCORD trial, and outcome reduction with initial glargine intervention (ORIGIN trial) showed severe hypoglycemia, serious adverse events, renal impairment, and increased mortality [5, 7, 8]. Thus, there is an urgent need to establish the new approach to treat DKD without hypoglycemia risk.

Extracellular matrix (ECM) accumulation, inducing mesangial expansion, is a critical process in the glomerular pathology induced by diabetic condition [9]. Type IV collagen (Col4) and smooth muscle actin are known as a common molecular marker of phenotypic changes of mesangial cells in DKD [10]. We have reported that the transforming growth factor-*β* (TGF-*β*)/Smad1 pathway transcriptionally regulates the expression of Col4 and *α*-smooth muscle actin (*α*SMA) [11–14]. Previous reports showed that endothelial to mesenchymal transition (EndMT) in the endothelial cells enhances the TGF-*β* signaling, leading to glomerulosclerosis in the diabetic kidney [15]. Recent reports suggest that supplementation with fish oil, which is a major source of the n-3 fatty acids eicosapentaenoic acid, has been suggested to prevent glomerulonephritis and type-2 diabetes [16, 17]. However, a mechanistic explanation regarding the action of ethyl eicosapentaenoate (EPA-E) on the glomerular cells is unknown.

In this study, we determined the effects of EPA-E on mesangial expansion and albuminuria in diabetic mice. Further, we investigated whether EPA-E could ameliorate adipocyte conditioned medium- or high glucose-induced EndMT in cultured endothelial cells.

## 2. Materials and Methods

### 2.1. Animal Experiments

The study was approved by an ethics committee or an institutional review board which is a part of Kindai University. All the animal protocols were approved by the Kindai University in accordance with the National Institutes of Health guidelines and ARRIVE guidelines (approval number: KAAG-26-010). We used male C57/BL6 mice (Shimizu, Kyoto, Japan). Diabetes was induced in 11-week-old C57/BL6 mice by intraperitoneal injection of streptozotocin (50 mg/kg body weight; Sigma, St Louis, MO) in 0.05 mol/l citrate buffer (pH 4.5) or citrate buffer for controls for 5 consecutive days. Nondiabetic mice (*n* = 8) were fed a control diet (MF diet, Oriental Yeast, Tokyo, Japan), and diabetic mice (*n* = 4) were fed a high-fat diet (60% from fat; HFD-60, Oriental Yeast, Tokyo, Japan). Two weeks after, some diabetic mice were fed with a high-fat diet were orally administrated EPA-E (Mochida Pharmaceutical, Tokyo, Japan) at a dose of 1000 mg/kg body weight/day in the diet for 19 weeks (*n* = 6) [18, 19]. The systolic, mean, and diastolic blood pressure were measured by the tail-cuff method (BP-98A; Softron, Tokyo, Japan). After 19 weeks of EPA-E treatment, kidney samples were collected for histological analysis, malondialdehyde assay, western blotting, and real-time PCR assay.

### 2.2. Intraperitoneal Glucose Tolerance Test

The intraperitoneal glucose tolerance test was performed after 12 weeks of the EPA administration (25 weeks of age). After 16 hours of fasting, the mice were intraperitoneally treated with glucose (2 g/kg body weight), which was followed by blood sampling from the tail vein at intervals of 0, 30, 60, 90, and 120 minutes. The blood glucose level was measured by the glucose analyzer (Sanwa Kagaku, Aichi, Japan).

### 2.3. Measurement of the Urinary Albumin and Creatinine

The urinary albumin and creatinine were measured using the Albuwell (Exocell Inc., PA, USA) and by creatinine colorimetric detection kit (Enzo Life Science, NY, USA), respectively. The urine samples were collected from the mice housed in individual metabolic cages. Urinary albumin and creatinine were measured at 11 weeks old (before treatment of EPA-E) and 28 weeks old (after 15 weeks of EPA-E treatment).

### 2.4. Plasma Triglyceride and Nonesterified Fatty Acid

After 19 weeks of EPA-E treatment, blood samples were collected for measurements of plasma triglyceride and nonesterified fatty acid. The plasma triglyceride and nonesterified fatty acid were measured using the LabAssay Triglyceride (FUJIFILM Wako Pure Chemical Corporation, Osaka, Japan) and LabAssay NEFA (FUJIFILM Wako Pure Chemical Corporation, Osaka, Japan), respectively.

### 2.5. Malondialdehyde Assay

Malondialdehyde in the renal cortex was measured using the TBARS assay kit (Cell Biolabs, San Diego, CA, USA), according to the manufacturer's instructions.

### 2.6. Histological Analysis

Kidney sections for light microscopy analysis were fixed in 4% paraformaldehyde phosphate buffer. Sections were stained with periodic acid-Schiff. Glomeruli were digitally photographed, and the images were imported to the ImageJ software (National Institutes of Health, Bethesda, MD, USA; https://imagej.nih.gov/ij/) and analyzed morphometrically [20]. For immunohistochemistry, the tissue sections were deparaffined using xylene and rehydrated through an ethanol series and PBS. The antigen retrieval was performed by microwave treatment, with Citrate buffer, pH 6. Endogenous peroxidase was blocked with 0.3% H_2_O_2_ in methanol for 30 min, followed by incubation with the G-Block (Genostaff, Tokyo, Japan) and avidin/biotin blocking kit (Vector, CA, USA). The sections were incubated with an anti-CD31 rabbit monoclonal antibody (Cell Signaling, MA, USA) at 4°C overnight. They were incubated with biotin-conjugated anti-rabbit Ig (Dako, CA, USA), for 30 min at room temperature, followed by the addition of peroxidase-conjugated streptavidin (Nichirei, Tokyo, Japan) for 5 min. The peroxidase activity was visualized using diaminobenzidine. The sections were counterstained with Mayer's Hematoxylin (MUTO, Tokyo, Japan), dehydrated, and then mounted with Malinol (MUTO).

### 2.7. 3T3-L1 Differentiation for Adipocyte

The mouse 3T3-L1 preadipocytes were cultured in DMEM (FUJIFILM Wako Pure Chemical Corporation, Osaka, Japan) containing 10% fetal bovine serum (FBS) at 37°C, 5% CO_2_. The differentiation for adipocytes was induced by a commercial kit (AdipoInducer Reagent, Takara Bio, Shiga, Japan). Two days postconfluency, 3T3-L1 preadipocytes were cultured using differentiation medium (DMEM containing 10 *μ*g/mL insulin, 2.5 *μ*M dexamethasone, and 0.5 mM 3-isobutyl-1methylxanthine) for 48 h. Then, the differentiation medium was replaced with a maintenance medium (DMEM containing 10 *μ*g/mL insulin). Thereafter, the maintenance medium was replaced every two days. After differentiation, the medium was collected on the 4^th^ and 8^th^ days, as the adipocyte-conditioned medium for stimulating the endothelial cells.

### 2.8. Oil Red O Stain

To evaluate the lipid accumulation of 3T3-L1 adipocyte, the Oil red O staining was performed. The cells were washed by PBS and fixed in 2.5% glutaraldehyde for 10 min. After washing, the cells were stained by Oil red O solution (FUJIFILM Wako Pure Chemical Corporation, Osaka, Japan) for 15 min. The stained cells were visualized under an optical microscope. Then, the Oil red O stained lipid was extracted by isopropanol and quantified by measuring the absorbance at 492 nm using a microplate reader (Thermo Fisher Scientific, MA, USA).

### 2.9. Endothelial Cell Culture

The cell line (bEnd.3) that was established from the mouse microvascular endothelial cells was used. EPA-E was dissolved in 100% ethanol to make stock solution. The cells were plated onto a 6-well plate and cultured in DMEM containing 10% FBS and 5.6 mM glucose. After reaching subconfluence, the medium was replaced by a 3T3-L1 adipocyte conditioned medium containing 5.6 mM glucose and 2% fatty acid-free BSA (FUJIFILM Wako Pure Chemical Corporation, Osaka, Japan) with or without a 200 *μ*M EPA-E (Mochida Pharmaceutical, Tokyo, Japan). The same amount of ethanol as EPA-E solution was used for the vehicle. After incubation for 48 h, the protein lysate was harvested for western blot analysis.

### 2.10. Adipocyte Conditioned Medium-Induced Cell Migration Assay

The cell culture inserts (Greiner Bio-One Co. Ltd, Tokyo, Japan) with an 8 *μ*m pore membrane were used for the cell migration assay as previously described with modifications [15, 21]. The endothelial cells were passaged in the upper chamber. Twenty-four hours after passage, the medium was changed to the 3T3-L1 adipocyte conditioned medium containing 5.6 mM glucose and 2% fatty acid-free BSA with or without of 200 *μ*M EPA-E. After 48 h, the nonmigratory cells were removed with a cotton swab. The migrated cells were stained with DAPI and counted. Five different areas were evaluated in each group.

### 2.11. High Glucose-Induced Endothelial Cell Migration

The cell culture inserts (Greiner Bio-One Co. Ltd, Tokyo, Japan) with 8 *μ*m pore membrane were used for cell migration assay. The endothelial cells were passaged in upper chamber. Twenty-four hours after passage, the medium was changed to low glucose (5.6 mM glucose and 19.4 mM mannitol) or high glucose medium (25 mM glucose) with or without of 50 *μ*M EPA-E. After 24 h, the nonmigratory cells were removed with a cotton swab. The migration cells were stained with DAPI and counted. Five different areas were evaluated in each group.

### 2.12. Western Blotting

*In vivo* experiments, protein samples were isolated from renal cortex. The protein lysates were separated by 10% SDS-polyacrylamide gels and blotted onto polyvinylidene fluoride (PVDF) membranes. After blocking, the membranes were incubated with anti-CD31 (Cell Signaling, MA, USA), anti-SM22*α* (Abcam, Cambridge, UK), anti-Erk1/2 (Cell Signaling, MA, USA), anti-pErk1/2 (Cell Signaling, MA, USA), anti-PAI-1 (Abcam, Cambridge, UK), TGF-*β* (Cell Signaling, MA, USA), anti-Snail (Cell Signaling, MA, USA), anti-TGF-*β* (Cell Signaling, MA, USA), and anti-*β*-actin (Cell Signaling, MA, USA) at 4°C overnight. The membranes were washed and incubated with horseradish peroxidase-conjugated secondary antibodies for 1 h at room temperature. The protein-antibody complex was detected using the ECL reagent (SuperSignalTM West Dura Extended Duration Substrate, Thermo Fisher Scientific, MA, USA), and the signals were detected using LAS 4000mini biomolecular imager (FUJIFILM, Tokyo, Japan). Membranes are cut prior to hybridization with antibodies, so these are not images of full-length blots.

### 2.13. RNA and MicroRNA Isolation and Real-Time PCR Assay

The total RNA was isolated from renal cortex using the RNeasy Mini Kit (Qiagen, German). The complementary DNA was synthesized using the SuperScript III reverse transcriptase (Invitrogen, Carlsbad, CA, USA). The real-time PCR was performed on a StepOne Plus real-time PCR system (Thermo Fisher Scientific, MA, USA) using the SYBR Green Master Mix (Applied Biosystems, Foster City, CA, USA). The expression levels were normalized to levels of GAPDH. PCR primer was as follows:

*Collagen IV*: GCC AAG TGTGCATGAGAAGA, AGCGGGGTGTGTTAGTTACG;

*TGF-β:* TGCTTCAGCTCCACAGAGAA, TGGTTGTAGAGGGCAAGGAC;

*PKCβ:* GGGATTCCAGTGTCAAGTCTGCT, AGGACTGGAGTACGTGTGGATCTT;

*p47phox:* ACCTTCATTCGCCATATCGCCCT, TTCTGTAGACCACCTTCTCCGACA;

*Nox2:* TGCAGCCTGCCTGAATTTCAACTG, AGATGTGCAATTGTGTGGATGGCG;

*Nox4*: GAACCTCAACTGCAGCCTGATC, CTTTTGTCCAACAATCTTCTTGTTCTC;

*GAPDH:* ATGTTCCAGTATGACTCCACTCACG, GAAGACACCAGTAGACTCCACGACA.

The primers for *Mm_miR-29b* and *Mm_let-7a* were from the miScript Primer Assay designed by Qiagen. The mature microRNA sequences were as follows: *Mm_miR-29b:* UAGCACCAUUUGAAAUCAGUGUU; *Mm_let-7a:* UGAGGUAGUAGGUUGUAUAGUU. All of the experiments were performed in triplicates, and *Hs_RNU6-2_1* (Qiagen) was used as an endogenous control for normalization.

### 2.14. Statistics

All statistical analyses were performed using Microsoft Excel (Microsoft Corporation, Redmond, WA, USA) and the add-in software Statcel 3 (OMS Publishing Inc., Saitama, Japan). The data are shown as the mean ± SD for each group. Statistical analysis was performed by one-way analysis of variance (ANOVA) post hoc Tukey—Kramer multiple comparisons, or Student's *t*-test. All results were considered statistically significant at *P* < 0.05.

## 3. Results

### 3.1. Characteristic of the Experimental Group

**Figure 1:**
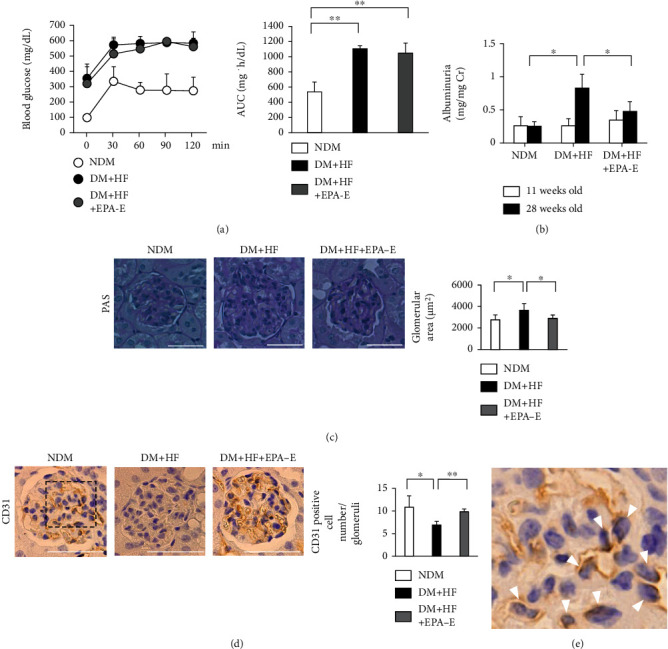
Effects of EPA-E on blood glucose, albuminuria, renal morphology, and immunohistochemical staining of CD31 in the experimental groups. (a) Intraperitoneal glucose tolerance tests were performed in the experimental groups. Time-course measurements of blood glucose is shown. The area under the curve is derived from the intraperitoneal glucose tolerance tests. (b) Albuminuria was measured by the Albuwell. (c) Representative light microscopic appearance of glomeruli (periodic acid-Schiff) for experimental groups. Morphometric analysis of the glomerular surface area. Bar = 50 *μ*m. (d) Representative immunohistochemistry of CD31 in the glomeruli. Sections were counterstained with hematoxylin solution. Bar = 50 *μ*m. (e) Enlarged glomerular image of the box in d. Positive nuclear staining for CD31 was localized in endocapillary area (white triangle). ^∗^*P* < 0.05. ^∗∗^*P* < 0.01. These data are expressed as means ± SD. NDM: nondiabetic mice; DM + HF: mice with STZ-induced diabetes were fed a high-fat diet; DM + HF + EPA − E: STZ-induced diabetic mice were fed a high-fat diet treated with EPA-E.

There was an increase in blood glucose by 3.2 ± 1.0-fold, kidney weight by 1.1 ± 0.4-fold, and albuminuria by 3.3 ± 0.8-fold in the diabetic mice fed a high-fat diet compared to the control mice after 5 months of diabetes. The final body weights of experimental groups did not change. Kidney weight per body weight increased by 1.1 ± 0.4-fold in diabetic mice compared to the control mice (Table 1). Systolic blood pressure was increased by 1.1 ± 0.1-fold compared to the control mice. Serum triglyceride and nonesterified fatty acid were increased by 1.4 ± 1.6- and 1.5 ± 1.6-fold higher, respectively (Table 2). The effects of diabetes and high-fat diet on the systemic insulin resistance studied by intraperitoneal glucose tolerance test showed a significant increase in glucose in a step-wise manner with maximum levels on diabetic mice fed a high-fat diet reached at 1 h with levels > 500 mg/dL (Figure 1(a)).

### 3.2. Effect of EPA-E on the Kidney of High Fat-Fed Diabetic Mice

Diabetic mice exhibited more albuminuria compared to the control mice by 3.3 ± 0.8-fold. Administration of EPA-E decreased albuminuria by 42 ± 18% (Figure 1(b)). The glomerular surface area was prominent in the diabetic mice fed a high-fat diet compared to the control mice (control mice; 2757 ± 444 *μ*m^2^ vs. diabetic mice; 3636 ± 613 *μ*m^2^, Figure 1(c)). Quantitative analysis of immunohistochemistry showed that treatment with EPA-E significantly decreased glomerular surface area by 21 ± 9% compared to the diabetic mice (Figure 1(c)). The positive cell number of CD31 in the glomeruli was decreased by 0.6 ± 0.1-fold in diabetic mice compared to the control mice and EPA-E treatment increased the positive cell number of CD31 by 43 ± 9% compared to the diabetic mice (Figure 1(d)).

**Figure 2:**
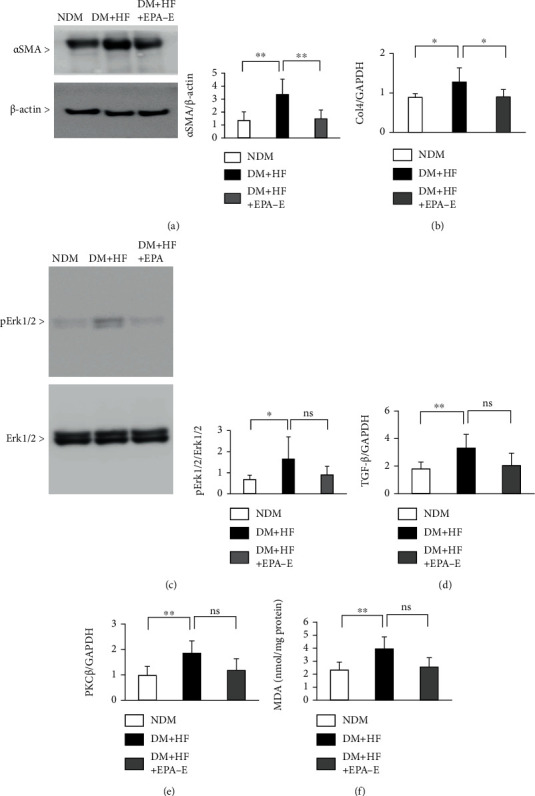
Effect of EPA-E on TGF-*β*, PKC*β*, and extracellular matrix in the experimental groups. (a) Immunoblot analysis of *α*SMA in the renal cortex of the nondiabetic mice, STZ-induced diabetic mice, and STZ-induced diabetic mice treated with EPA-E. (b) *Type 4 collagen* mRNA expression in the renal cortex of the experimental groups. (c) Immunoblot analysis of phosphor-Erk1/2 in the renal cortex of the experimental groups. (d) and (e) TGF-*β* (d) and PKC*β* (e) mRNA expression in the renal cortex of the experimental groups. (f) Malondialdehyde is measured by the thiobarbituric acid reactive substance assay. ^∗^*P* < 0.05. ^∗∗^*P* < 0.01. ns: not significant. These data are expressed as means ± SD. NDM: nondiabetic mice; DM + HF: mice with STZ-induced diabetes were fed a high-fat diet; DM + HF + EPA − E: STZ-induced diabetic mice were fed a high-fat diet treated with EPA-E. Regarding immunoblot, membranes are cut prior to hybridization with antibodies, so these are not images of full-length blots.

Expression of *α*SMA in the renal cortex was increased by 2.5 ± 0.9-fold in the diabetic mice and EPA-E treatment decreased its expression by 56 ± 20% (Figure 2(a)). Diabetes increased the *Col4* mRNA levels by 1.4 ± 0.4-fold. The EPA-E treatment decreased the levels by 30 ± 15% (Figure 2(b)). Phosphorylation of Erk1/2 was increased in diabetic mice by 2.4 ± 1.5-fold compared with control mice (Figure 2(c)). Although the results are not significant, there was a trend for a beneficial effect of EPA-E; it decreased the expression of p-Erk1/2 by 45 ± 25% (Figure 2(c)). TGF-*β* and PKC*β* mRNA were increased in diabetic mice by 1.8 ± 0.6-fold and 1.9 ± 0.5-fold, respectively (Figures 2(d) and 2(e)). There was a similar trend for a beneficial effect of EPA-E in these mRNA levels. However, no statistically significant differences were found between these groups (Figures 2(d) and 2(e)).

### 3.3. Oxidative Stress in Renal Cortex

We also characterized the markers of oxidative stress, which could contribute to developing DKD [22–25]. Malondialdehyde (MDA), as measured by thiobarbituric acid reactive substance assay, increased in renal cortex of diabetic mice by 1.7 ± 0.4-fold, which was decreased by EPA-E treatment 35 ± 18% (Figure 2(f)). Diabetes increased the mRNA expression of *p47phox, Nox2*, and *Nox4* in the renal cortex by 6.2 ± 4.6-fold, 3.6 ± 2.7-fold, and 1.3 ± 0.1-fold, respectively (Supplementary Figures1a, 1b, and 1c). Similarly, EPA-E decreased these expressions by 62 ± 50%, 40 ± 41%, and 16 ± 15%, respectively. However, there were no statistically significant differences between these groups (Supplementary Figures1a, 1b, and 1c).

### 3.4. Adipocyte Differentiation Promotes EndMT and EPA Inhibits Its Effect

**Figure 3:**
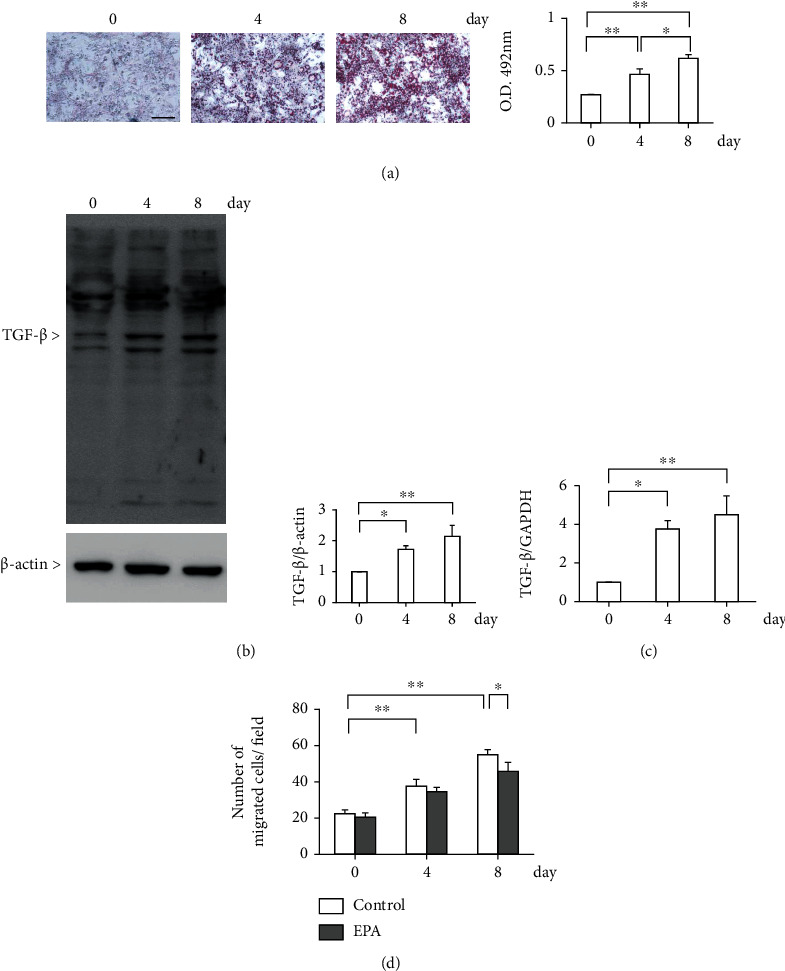
Adipocyte differentiation promotes End-MT and EPA-E inhibits its effect. (a) The 3T3-L1 cells were cultured in differentiation medium for 8 days. Cells that were differentiated into adipocytes were stained with Oil red O staining. Oil red O staining was evaluated by measuring the absorbance of the eluate. Bar = 100 *μ*m. (b) and (c) Protein expression (b) and mRNA expression (c) of TGF-*β* in the 3T3L1 preadipocyte cells. (d) End-MT was evaluated using the Boyden chamber assay. ^∗^*P* < 0.05. ^∗∗^*P* < 0.01. These data are expressed as means ± SD.

**Figure 4:**
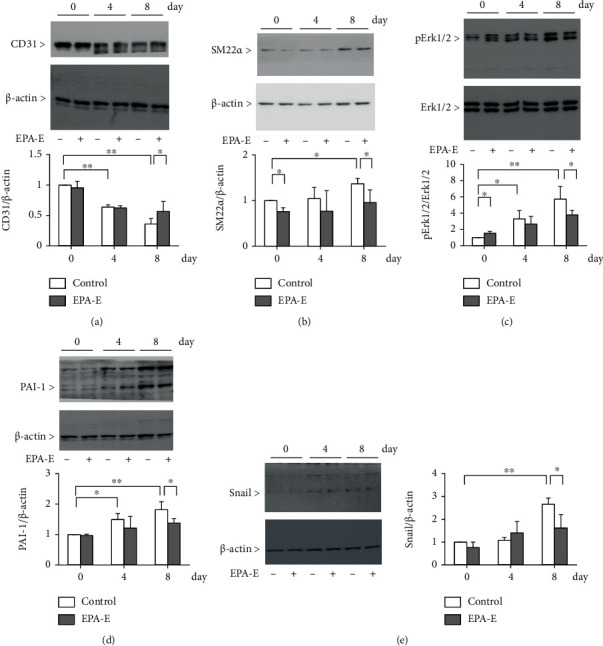
EPA-E decreased the adipogenic differentiation medium induced-End-MT. Immunoblot analysis of CD31, SM22*α*, phosphorylation of Erk1/2, PAI-1, and Snail. Endothelial cells were incubated in the adipocyte conditioned medium with or without EPA-E (200 *μ*M). (a) CD31. (b) SM22*α*. (c) Phosphorylation of Erk1/2. (d) PAI-1. (e) Snail. ^∗^*P* < 0.05. ^∗∗^*P* < 0.01. These data are expressed as means ± SD. Regarding immunoblot, membranes are cut prior to hybridization with antibodies, so these are not images of full-length blots.

To determine whether a high-fat condition can cause EndMT due to the process of adipogenic differentiation, the 3T3-L1 preadipocyte cells were used. As shown in our results, Oil red O staining increased gradually from 0 to 8 days (Figure 3(a)). Consistent with this, the protein levels of TGF-*β* were also gradually increased in 3T3L1 preadipocyte cells (1.7 ± 0.1-fold in 4 days and 2.1 ± 0.4-fold in 8 days compared to 0 days, respectively, Figure 3(b)). Expression of TGF-*β* mRNA was also increased in the same manner (3.8 ± 0.6-fold in 4 days and 4.5 ± 1.5-fold in 8 days compared to 0 days, respectively, Figure 3(c)). EndMT was evaluated with the Boyden chamber assay. The addition of the conditioned medium from adipocyte culture was found to promote EndMT by 2.5 ± 0.1-fold in 8 days compared to 0 days. Treatment with EPA-E decreased the End-MT in 8 days by 17 ± 9% (Figure 3(d)). In addition, high glucose condition induced migration of endothelial cells by 2.3 ± 0.3-fold when compared to low glucose condition. The addition of EPA-E decreased the EndMT by 27 ± 9% (Supplementary Figure 2). It is reported that TGF-*β* induced the suppression of CD31 with concomitant upregulation of SM22*α* in endothelial cells, suggesting that TGF-*β* increases EndMT [26]. We have checked whether adipogenic differentiation medium induces EndMT. Adipogenic differentiation medium decreased expression of endothelial cell marker CD31 by 64 ± 9%, while the protein expression of SM22*α* was increased by 1.4 ± 0.1-fold. EPA-E partially normalized CD31 expression by 58 ± 46% and decreased SM22*α* expression by 30 ± 20% (Figures 4(a) and 4(b)). As activation of the Erk1/2-PAI-1 pathway plays a significant role in developing DKD following EndMT [20, 27], this pathway was checked in the endothelial cells. Phosphorylation of Erk1/2 and plasminogen activator inhibitor-1 (PAI-1) protein expression was increased by 5.7 ± 1.6-fold and 1.8 ± 0.3-fold in adipogenic differentiation medium, respectively. Furthermore, EPA-E decreased these expressions by 34 ± 10% and 24 ± 8%, respectively (Figures 4(c) and 4(d)). Snail, a key regulator of TGF-*β* expression was also increased by 2.7 ± 0.3-fold. The addition of EPA-E decreased this expression by 39 ± 22% (Figure 4(e)).

To characterize the effects of adipocyte medium on TGF-*β* action in EndMT, we tested the effect of LY364947, selective inhibitor of TGF-*β*RI, in endothelial cells. The addition of the conditioned medium from adipocyte culture was found to decreases in the protein expression of CD31 by 33 ± 0.1% when compared to control. LY364947 reversed its expression only by 2.1 ± 0.1 and 6.5 ± 0.3% (LY364947; 1 *μ*M and 10 *μ*M, respectively, Supplementary Figure 3).

### 3.5. Expression of Mm_miR-29b and Mm_let-7a in the Renal Cortex of Diabetic Mice

*Mm_miR-29b* and *Mm_let-7a* exhibit renal protective effects and act as a negative regulator of the EndMT via inhibition of TGF-*β* signaling, while these expressions decrease in renal injury [28–30]. Thus, the expressions of these miRNAs were evaluated in the renal cortex. In the renal cortex of diabetic mice, the level of *Mm_let-7a* was significantly decreased by 41 ± 24% compared to the control mice (Supplementary Figure 4a). In contrast, there were no statistically significant differences in *Mm_miR-29b* expression between these groups (Supplementary Figure 4b). Next, we tested whether the administration of EPA-E affects *Mm_miR-29b* and *Mm_let-7a*. However, EPA-E did not affect any of these (Supplementary Figures 4a and 4b).

## 4. Discussion

In the present study, we have demonstrated that progression of EndMT may be occurred in the glomeruli of diabetic mice, leading to DKD. Furthermore, EPA-E was found to improve the mesangial expansion and albuminuria through the trend for inhibition of EndMT and TGF-*β*-mediated renal fibrosis signaling.

Extracellular matrix, such as type I and IV collagen, or *α*-SMA, which is regulated by TGF-*β* and bone morphogenetic protein 4 (BMP4), is increased in the glomeruli of patients with type-2 diabetes and one of the important features of DKD [31]. Notably, the expression of TGF-*β*-induced EndMT in the endothelial cells is recognized in diabetic conditions, which could be suppressed by the linagliptin-mediated microRNA 29 induction [15]. However, we used bEnd.3 which is derived from the mouse microvascular endothelial cells. Thus, the results of our results may not reflect the effects on renal glomeruli.

In the Reduction of Cardiovascular Events with Icosapent Ethyl-Intervention (REDUCE-IT) and the Japan EPA Lipid Intervention Study (JELIS), noted a tendency toward the use of high-dose n-3 polyunsaturated fatty acids (PUFA), EPA significantly reduced the risk of cardiovascular diseases [32, 33]. Although not directly assessing patients with DKD, many patients in REDUCE-IT had diabetes and thus the effects of EPA treatment in diabetic models may be relevant to these other studies.

DHA-E reduces blood pressure [34, 35], heart rate [36, 37], and platelet aggregation [38, 39] more efficiently than EPA-E. DHA-E has a longer carbon chain and additional double bonds than EPA-E, it undergoes rapid isomerization, which increases membrane fluidity rather than stability, resulting in a rapid decrease in antioxidant capacity [40–42]. Hence, the effects of EPA-E and DHA-E may be different. Further study will be needed to clarify.

It is reported that plasma level of EPA-E is 62 *μ*g/mL when the rat was treated with EPA-E at 1000 mg/kg/day for 4 weeks, which is less than in humans given a clinical dose of EPA-E (1800 mg/day) for 3 months (143 *μ*g/mL) [18, 19]. Thus, 1000 mg/kg/day is considered to be an appropriate dose for our *in vivo* experiments of the pharmacological effects of EPA-E in mice.

The activation of PKC*β* has been identified to lead to the inhibition of glomeruli endothelial cell function [20, 43]. The diminution of the endothelial nitric oxide synthase activation and endothelial dysfunction in the glomeruli could contribute to the loss of antioxidative and inflammatory effects of nitric oxide [22, 43, 44] . The phosphorylation of Erk and increase in PKC*β* are recognized in diabetic mice which is consistent with the previous report [20, 45]. The increase in phosphorylation of Erk in the diabetic condition is presumably because of the activation of PKC*β*, which can increase MAPK [20].

Reflecting on the emergence of glomeruli endothelial dysfunction and EndMT, the number of CD31-positive cells was decreased, and *α*SMA expression was increased in the glomeruli of the diabetic mice. EndMT is characterized by the reduced expression of the endothelial markers, such as CD31 and VE-cadherin, and increases the expression of mesenchymal markers, such as SM22*α* and *α*SMA [46]. Thus, cell adhesion molecules are decreased, while cell migration ability is increased in EndMT. It has been reported that EndMT is controlled by a variety of stimuli, including TGF-*β*, high glucose, oxidative stress, TNF-*α*, and IL-1*β* [47]. However, the relationship between adipocyte differentiation and EndMT has not been clear. Our results indicate that there was a decrease in CD31 expression and an increase in SM22*α* expression in the cultured endothelial cells during adipocyte hypertrophic differentiation. Further, cell migration assay in our study revealed that both adipocyte conditioned medium and high glucose could induce endothelial cell migration, leading to EndMT. However, there are few reports regarding cell migration assay in diabetic condition [48]. Although convincing, there is a limitation in this point.

TGF-*β* induces EndMT through the Smad, MEK/ERK, PI3K, and p38MAPK signaling pathways and increases the expression of Snail, which is the cell adhesion suppressing transcription factor. We have previously reported that TGF-*β*/BMP4-Smad1 pathway upregulates the expression of Col4 and *α*SMA [11, 13, 31]. Further, the inhibition of phosphorylation of Erk1/2 prevents the TGF-*β*-induced EndMT via the suppression of Snail expression [49]. TGF-*β* regulated-Snail decreases the endothelial cell characteristics and, counterintuitively, increases the expression of mesenchymal markers such as *α*SMA and SM22*α* [50, 51]. However, our data may not be enough to affirm that EPA-E acts through EndMT and TGF-*β*-mediated renal fibrosis because no statistically significant difference was found. This problem may have occurred owing to the small samples. Therefore, further study will be needed to clarify this.

Our results indicated that suppression of TGF-*β* alone is not sufficient to suppress End-MT. As shown in our results, TGF-*β* suppression by EPA-E was partial, while EPA-E completely reduced snail expression, which is more involved in End-MT. Not only TGF-*β* signaling but other signals such as notch signaling are involved in the increase of snail [52, 53]. Thus, EPA-E-induced reduction of snail expression may be the result of strong suppression of signals other than TGF-*β* signaling. Previous reports indicated that 3T3-L1 adipocyte secretes a variety of adipokines and lipids, resulting in increases in TGF-*β* [54, 55]. Therefore, the secretion of adipokines and other factors induced by the differentiation process of 3T3-L1cells into adipocytes could give rise to EndMT.

As we have confirmed, the vascular endothelial- (VE-) cadherin is an accurately endothelial-specific adhesion molecule located at the junctions between the endothelial cells [20, 56, 57]. The cancer cell-conditioned medium decreased the expression of VE-cadherin in the endothelial cells by the binding of Snail to the VE-cadherin promoter [58]. Our data showed that EPA-E suppressed the expression of Snail and the phosphorylation of Erk1/2 by the adipocyte conditioned medium. Thus, EPA-E regulated the expression of CD31 and SM22*α* by suppressing the phosphorylation of Erk1/2 and TGF-*β*-Snail signaling.

There is substantial evidence indicating that the reactive oxygen species (ROS) is increased in the retina, kidney, and endothelial cells either when exposed to diabetic conditions or in the diabetic rodent model [59]. Further, ROS regulates the TGF-*β*-induced expression of *α*SMA and Nox4 in human cardiac fibroblasts. In contrast, the knockdown of *Nox4* with siRNA reduced the oxidative stress and expression of *α*SMA by TGF-*β* [60]. Our data showed an increase in the MDA levels, expression of *p47phox*, *Nox2*, and *Nox4* mRNAs in the diabetic kidney, although the increase was modest in the EPA-E-treated mice compared to the control mice. The administration of EPA-E has been found to decrease albuminuria without affecting glycemic control, serum lipid level, and blood pressure in patients with type-2 diabetes [61]. Previous reports indicate that the intraperitoneal injection of EPA-E ameliorated the mesangial matrix expansion and decreased the phosphorylation of Erk1/2 in KKAy/Ta mice [62]. EPA-E also suppressed the diabetes-induced upregulation of MCP-1 and TGF-*β* expression, along with the reduction of MDA [63].

The results obtained from the conditioned medium from the cultured adipocyte demonstrated that the PKC activation increased the phosphorylation of Erk1/2, which leads to the activation of PAI-1. In contrast, EPA-E inhibited the increase in the phosphorylation of Erk1/2, PAI-1 expression in the endothelial cells, and the ability of the endothelial cell to migrate. PAI-1 increased the ECM accumulation through inhibition of proteolysis and promotion of ECM synthesis by TGF-*β* in the diabetic kidney, while inhibition of PAI-1 decreased ECM accumulation with the suppression of Col4 expression [64, 65]. PAI-1 also promoted endothelial cell migration through inhibition of integrin-mediated cell adhesion [66]. Our results indicate that EPA-E not only suppresses EndMT but may also directly the signal to increase extracellular matrix.

MicroRNAs (miRNAs) are well known for their regulatory effects on several diseases such as diabetes, cancer cells, and renal fibrosis [15, 67, 68]. The differential miRNA expression indicated a role of altered miRNA in the pathogenesis of the renal disease [69, 70]. *Mm_miR-29b* is downstream of Smad3 and can inhibit the upstream TGF-*β*-Smad3 signaling by the *Mm_miR-29b*-regulated negative feedback, decreasing the type I and III collagen [28]. Similarly, the *Mm_let-7* family including *Mm_let-7a* inhibits the TGF-*β* signaling in renal fibrosis [29, 71, 72]. In our study, both *Mm_miR-29b* and *Mm_ let-7a* were reduced in diabetic mice, but the difference was not statistically significant; *Mm_ let-7a* was in fact significantly reduced in diabetic mice, but *Mm_miR-29b* was not significantly reduced as indicated in supplementary figure 2. Furthermore, the increase in these miRNAs upon EPA-E treatment was not evident. Thus, EndMT inhibition by EPA-E treatment may result from the direct inhibition of the PKC*β*/TGF-*β*/PAI-1 signaling, but not via miRNAs.

## 5. Conclusions

In summary, our study has identified the mechanism by which EPA-E can protect the glomerular endothelial cells by inhibiting EndMT followed by the PKC*β*/TGF-*β*/PAI-1 signaling. Further, we have demonstrated that these inhibitory effects by EPA-E regulated independently of the miRNAs. These findings indicate that EPA-E has the potential to impart renal protection by regulating EndMT in DKD.

## Figures and Tables

**Table 1:** General characteristics of the experimental groups.

	NDM	DM + HF	DM + HF + EPA − E
Number	8	4	6
Bodyweight (g)			
10-week old	25.1 ± 2.0	24.5 ± 3.9	24.3 ± 2.7
32-week old	34.7 ± 4.0#	33.0 ± 9.9	33.8 ± 5.8
rKW/BW (g/100 g BW)	1.06 ± 0.18	1.19 ± 0.38	1.13 ± 0.16
Blood glucose (mg/dL)	98.5 ± 14.9	314 ± 100^∗^	274 ± 92^∗^

NDM: nondiabetic mice; DM + HF: mice with STZ-induced diabetes fed a high-fat diet; DM + EPA − E: STZ-induced diabetic mice fed a high-fat diet treated with EPA-E; EPA-E: ethyl eicosapentaenoate; rKW/BW: right kidney weight/body weight. Data expressed as means ± SD. ^∗^*P* < 0.05 vs. NDM, #*P* < 0.01 vs. NDM 10-week old.

**Table 2:** Metabolic characteristics of experimental groups.

	NDM	DM	DM + EPA − E
Number	8	3	4
TG (mg/dL)	85.3 ± 23.4	123 ± 140	68.9 ± 26.2
NEFA (mEq/L)	0.52 ± 0.13	0.75 ± 0.83	0.33 ± 0.16
SBP (mmHg)	113 ± 8	126 ± 1^∗^	113 ± 6

NDM: nondiabetic mice; DM: mice with STZ-induced diabetes; DM + EPA − E: STZ-induced diabetic mice treated with EPA-E; EPA-E: ethyl eicosapentaenoate; TG: triglyceride; NEFA: nonesterified fatty acid; SBP: systolic blood pressure. Data expressed as means ± SD. ^∗^*P* < 0.05 vs. NDM. Blood sampling and blood pressure measurement could not be performed on all mice due to their conditions.

## Data Availability

The datasets generated during and/or analyzed during the current study are available from the corresponding author on reasonable request.

## Supplementary Materials

Supplementary Materials Supplementary Figure 1: evaluation of the oxidative stress in the renal cortex. (a)−(c) Characterization of the changes in the mRNA levels of p47phox (a), Nox2 (b), and Nox4 (c) expression in the renal cortex of the nondiabetic mice, STZ-induced diabetic mice, and STZ-induced diabetic mice treated with EPA-E. ^∗^*P* < 0.05. ns: not significant. These data are expressed as mean ± SD. NDM: nondiabetic mice; DM + HF: mice with STZ-induced diabetes were fed a high-fat diet; DM + HF + EPA − E: STZ-induced diabetic mice were fed a high-fat diet treated with EPA-E. Supplementary Figure 2: high glucose promotes End-MT and EPA-E inhibits its effect. End-MT was evaluated using the Boyden chamber assay. The endothelial cells were passaged in upper chamber. Twenty-four hours after passage, the medium was changed to low glucose (5.6 mM glucose and 19.4 mM mannitol) or high glucose medium (25 mM glucose) with or without of 50 *μ*M EPA-E. The migration cells were stained with DAPI and counted. ^∗^*P* < 0.05. ^∗∗^*P* < 0.01. These data are expressed as mean ± SD. Supplementary Figure 3: effect of selective inhibitor of TGF-*β*R1, LY36497 on CD31 expression in endothelial cells. Immunoblot analysis of CD3l. Endothelial cells were incubated in the adipocyte conditioned medium 1 *µ*M or 10 *µ*M LY36497. ^∗^*P* < 0.05. ns: not significant. These data are expressed as mean ± SD. Regarding immunoblot, membranes are cut prior to hybridization with antibodies, so these are not images of full-length blots. Supplementary Figure 4: Mm_miR-29b and Mm_let-7a reveal trends of suppression in the renal cortex. (a) and (b) miRNAs alteration in the renal cortex of the renal cortex of the nondiabetic mice, STZ-induced diabetic mice, and STZ-induced diabetic mice treated with EPA-E. (a) Mm_miR-29b. (b) Mm_let-7a. ^∗^*P* < 0.05. ns: not significant. These data are expressed as mean ± SD. NDM: nondiabetic mice; DM + HF: mice with STZ-induced diabetes were fed a high-fat diet; DM + HF + EPA − E: STZ-induced diabetic mice were fed a high-fat diet treated with EPA-E.
