# Supplementary figures and images for: Protective Effects of Eicosapentaenoic Acid on the Glomerular Endothelium via Inhibition of EndMT in Diabetes
Source: J Diabetes Res. 2021 Dec 24;2021:2182225. doi: 10.1155/2021/2182225 (PMC8720008; doi:10.1155/2021/2182225)

Supplementary Figure 1

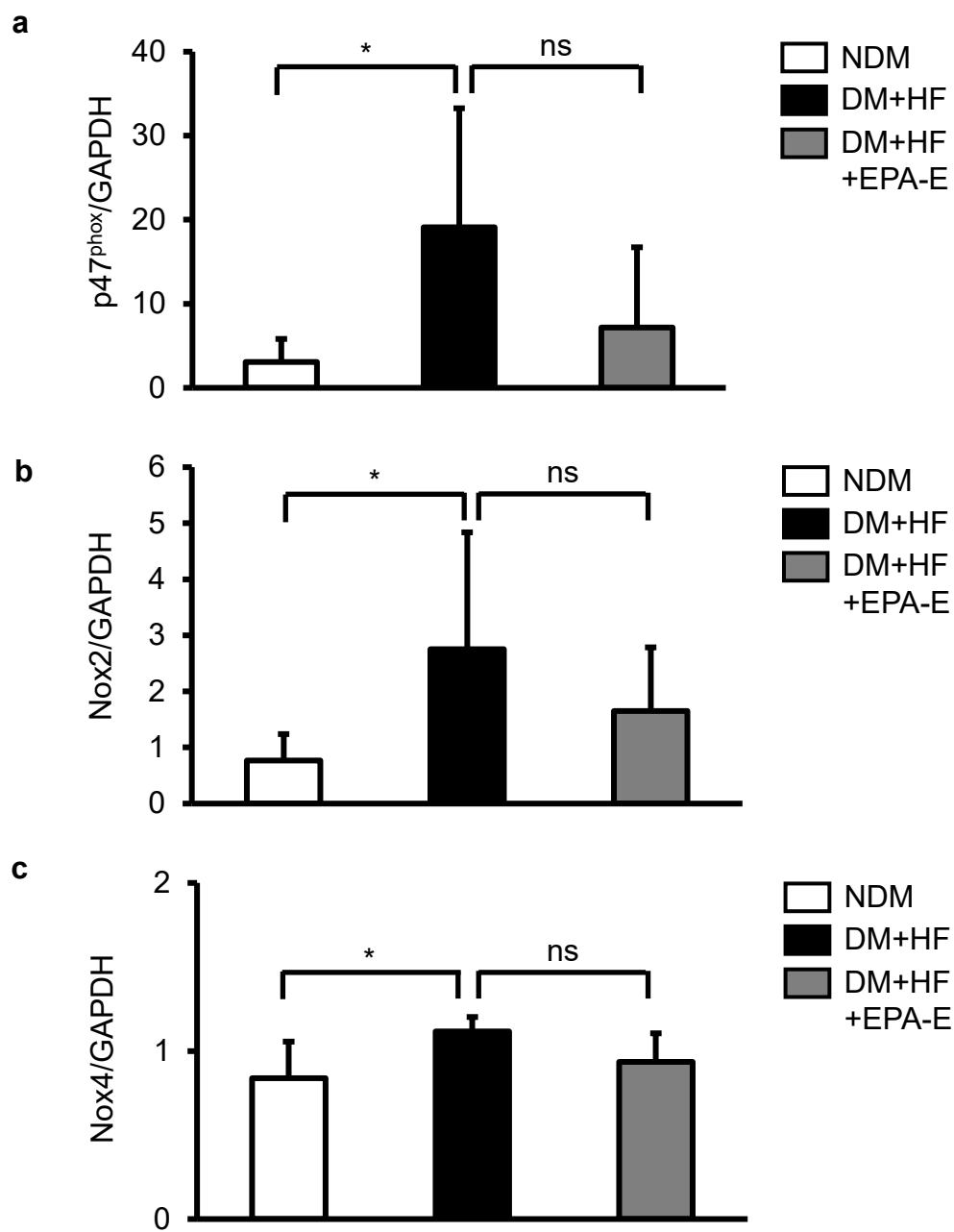

Supplement: Supplementary Materials — Supplementary Figure 1: evaluation of the oxidative stress in the renal cortex. (a)−(c) Characterization of the changes in the mRNA levels of p47phox (a), Nox2 (b), and Nox4 (c) expression in the renal cortex of the nondiabetic mice, STZ-induced diabetic mice, and STZ-induced diabetic mice treated with EPA-E. ∗P < 0.05. ns: not significant. These data are expressed as mean ± SD. NDM: nondiabetic mice; DM + HF: mice with STZ-induced diabetes were fed a high-fat diet; DM + HF + EPA − E: STZ-induced diabetic mice were fed a high-fat diet treated with EPA-E. Supplementary Figure 2: high glucose promotes End-MT and EPA-E inhibits its effect. End-MT was evaluated using the Boyden chamber assay. The endothelial cells were passaged in upper chamber. Twenty-four hours after passage, the medium was changed to low glucose (5.6 mM glucose and 19.4 mM mannitol) or high glucose medium (25 mM glucose) with or without of 50 μM EPA-E. The migration cells were stained with DAPI and counted. ∗P < 0.05. ∗∗P < 0.01. These data are expressed as mean ± SD. Supplementary Figure 3: effect of selective inhibitor of TGF-βR1, LY36497 on CD31 expression in endothelial cells. Immunoblot analysis of CD3l. Endothelial cells were incubated in the adipocyte conditioned medium 1 µM or 10 µM LY36497. ∗P < 0.05. ns: not significant. These data are expressed as mean ± SD. Regarding immunoblot, membranes are cut prior to hybridization with antibodies, so these are not images of full-length blots. Supplementary Figure 4: Mm_miR-29b and Mm_let-7a reveal trends of suppression in the renal cortex. (a) and (b) miRNAs alteration in the renal cortex of the renal cortex of the nondiabetic mice, STZ-induced diabetic mice, and STZ-induced diabetic mice treated with EPA-E. (a) Mm_miR-29b. (b) Mm_let-7a. ∗P < 0.05. ns: not significant. These data are expressed as mean ± SD. NDM: nondiabetic mice; DM + HF: mice with STZ-induced diabetes were fed a high-fat diet; DM + HF + EPA − E: STZ-induced diabetic mice were fed a [file 2182225.f1.zip › 2182225.f1/supple 1 6.16.2021.pdf]

Supplementary Figure2

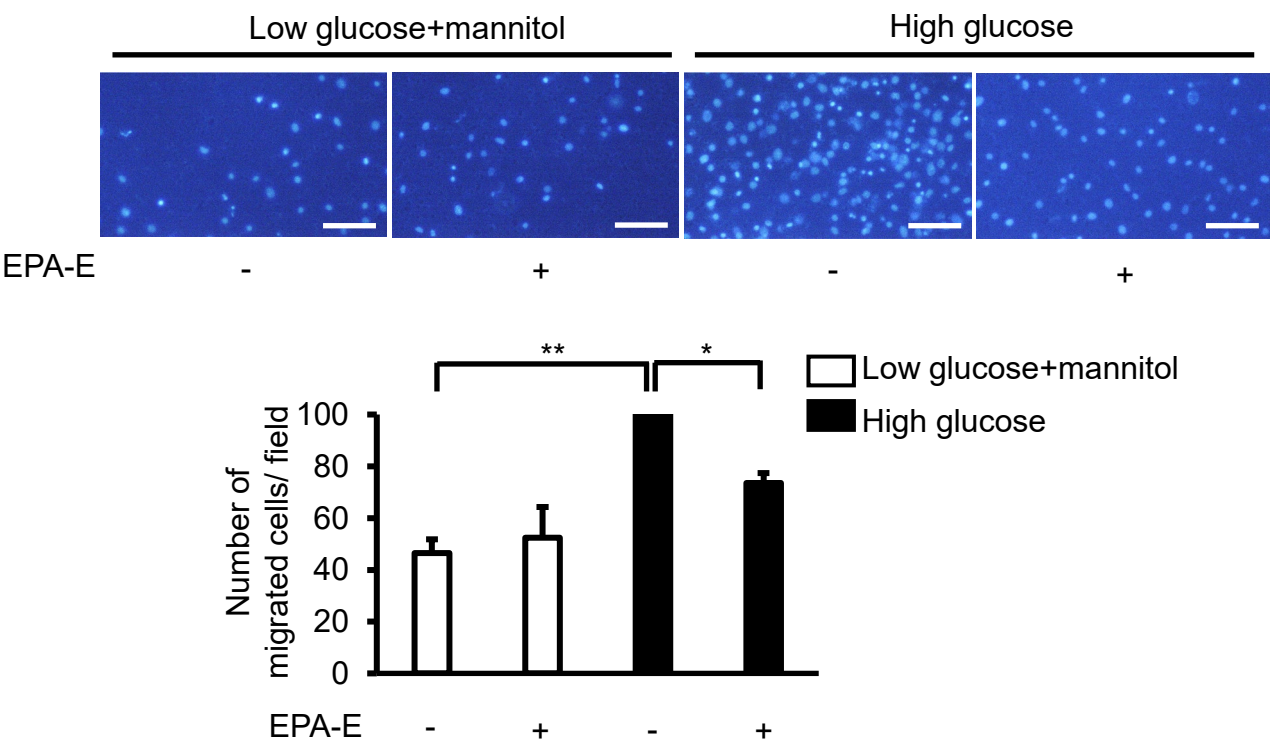

Supplement: Supplementary Materials — Supplementary Figure 1: evaluation of the oxidative stress in the renal cortex. (a)−(c) Characterization of the changes in the mRNA levels of p47phox (a), Nox2 (b), and Nox4 (c) expression in the renal cortex of the nondiabetic mice, STZ-induced diabetic mice, and STZ-induced diabetic mice treated with EPA-E. ∗P < 0.05. ns: not significant. These data are expressed as mean ± SD. NDM: nondiabetic mice; DM + HF: mice with STZ-induced diabetes were fed a high-fat diet; DM + HF + EPA − E: STZ-induced diabetic mice were fed a high-fat diet treated with EPA-E. Supplementary Figure 2: high glucose promotes End-MT and EPA-E inhibits its effect. End-MT was evaluated using the Boyden chamber assay. The endothelial cells were passaged in upper chamber. Twenty-four hours after passage, the medium was changed to low glucose (5.6 mM glucose and 19.4 mM mannitol) or high glucose medium (25 mM glucose) with or without of 50 μM EPA-E. The migration cells were stained with DAPI and counted. ∗P < 0.05. ∗∗P < 0.01. These data are expressed as mean ± SD. Supplementary Figure 3: effect of selective inhibitor of TGF-βR1, LY36497 on CD31 expression in endothelial cells. Immunoblot analysis of CD3l. Endothelial cells were incubated in the adipocyte conditioned medium 1 µM or 10 µM LY36497. ∗P < 0.05. ns: not significant. These data are expressed as mean ± SD. Regarding immunoblot, membranes are cut prior to hybridization with antibodies, so these are not images of full-length blots. Supplementary Figure 4: Mm_miR-29b and Mm_let-7a reveal trends of suppression in the renal cortex. (a) and (b) miRNAs alteration in the renal cortex of the renal cortex of the nondiabetic mice, STZ-induced diabetic mice, and STZ-induced diabetic mice treated with EPA-E. (a) Mm_miR-29b. (b) Mm_let-7a. ∗P < 0.05. ns: not significant. These data are expressed as mean ± SD. NDM: nondiabetic mice; DM + HF: mice with STZ-induced diabetes were fed a high-fat diet; DM + HF + EPA − E: STZ-induced diabetic mice were fed a [file 2182225.f1.zip › 2182225.f1/supple 2 6.16.2021.pdf]

Supplementary Figure 3

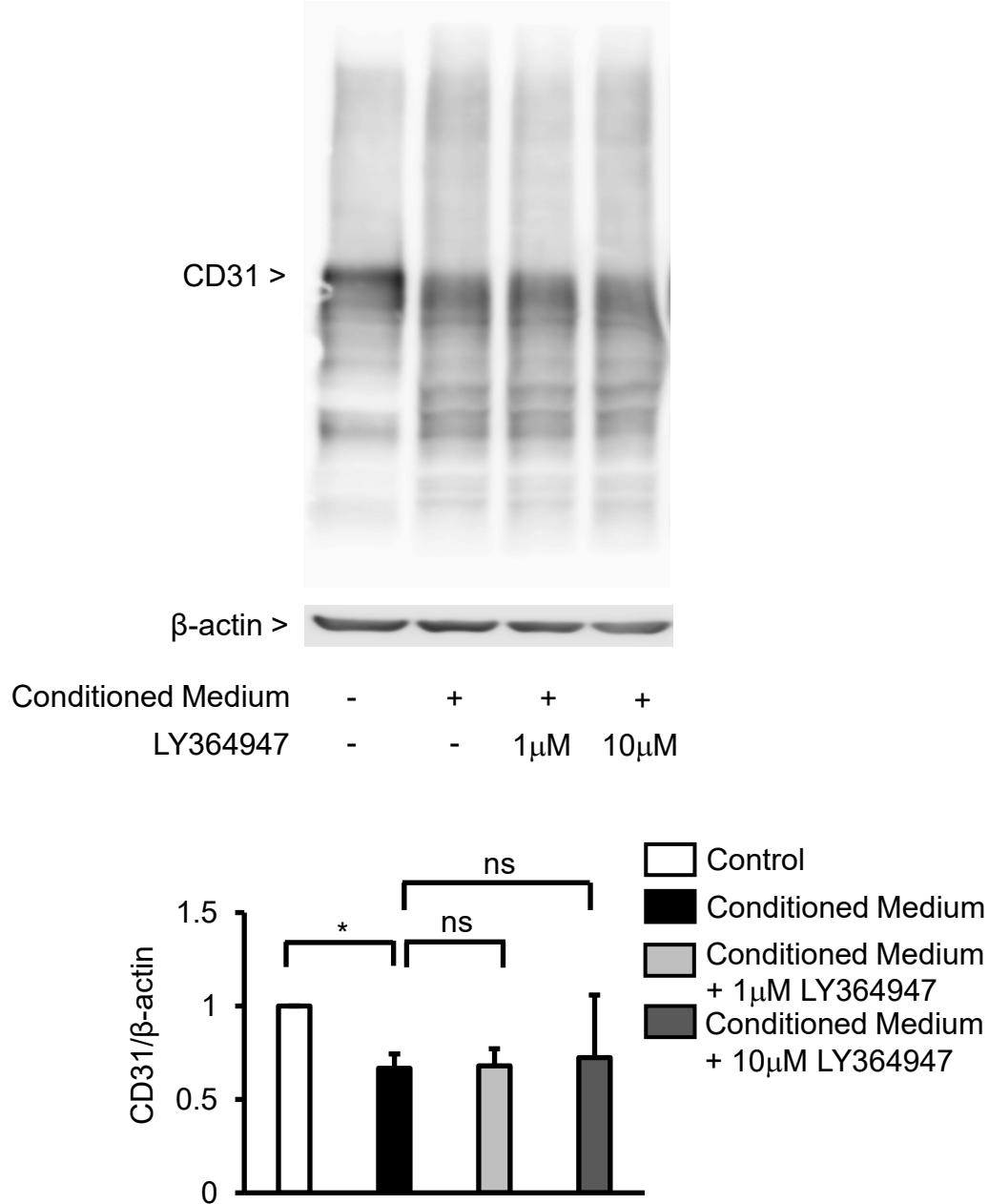

Supplement: Supplementary Materials — Supplementary Figure 1: evaluation of the oxidative stress in the renal cortex. (a)−(c) Characterization of the changes in the mRNA levels of p47phox (a), Nox2 (b), and Nox4 (c) expression in the renal cortex of the nondiabetic mice, STZ-induced diabetic mice, and STZ-induced diabetic mice treated with EPA-E. ∗P < 0.05. ns: not significant. These data are expressed as mean ± SD. NDM: nondiabetic mice; DM + HF: mice with STZ-induced diabetes were fed a high-fat diet; DM + HF + EPA − E: STZ-induced diabetic mice were fed a high-fat diet treated with EPA-E. Supplementary Figure 2: high glucose promotes End-MT and EPA-E inhibits its effect. End-MT was evaluated using the Boyden chamber assay. The endothelial cells were passaged in upper chamber. Twenty-four hours after passage, the medium was changed to low glucose (5.6 mM glucose and 19.4 mM mannitol) or high glucose medium (25 mM glucose) with or without of 50 μM EPA-E. The migration cells were stained with DAPI and counted. ∗P < 0.05. ∗∗P < 0.01. These data are expressed as mean ± SD. Supplementary Figure 3: effect of selective inhibitor of TGF-βR1, LY36497 on CD31 expression in endothelial cells. Immunoblot analysis of CD3l. Endothelial cells were incubated in the adipocyte conditioned medium 1 µM or 10 µM LY36497. ∗P < 0.05. ns: not significant. These data are expressed as mean ± SD. Regarding immunoblot, membranes are cut prior to hybridization with antibodies, so these are not images of full-length blots. Supplementary Figure 4: Mm_miR-29b and Mm_let-7a reveal trends of suppression in the renal cortex. (a) and (b) miRNAs alteration in the renal cortex of the renal cortex of the nondiabetic mice, STZ-induced diabetic mice, and STZ-induced diabetic mice treated with EPA-E. (a) Mm_miR-29b. (b) Mm_let-7a. ∗P < 0.05. ns: not significant. These data are expressed as mean ± SD. NDM: nondiabetic mice; DM + HF: mice with STZ-induced diabetes were fed a high-fat diet; DM + HF + EPA − E: STZ-induced diabetic mice were fed a [file 2182225.f1.zip › 2182225.f1/supple 3 6.16.2021.pdf]

Supplementary Figure 4

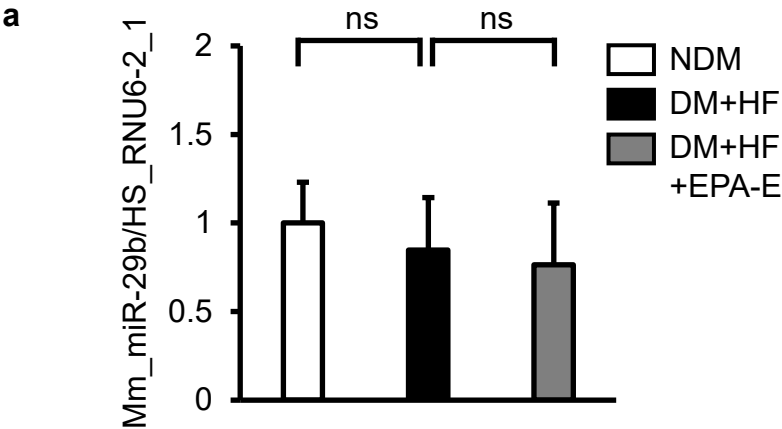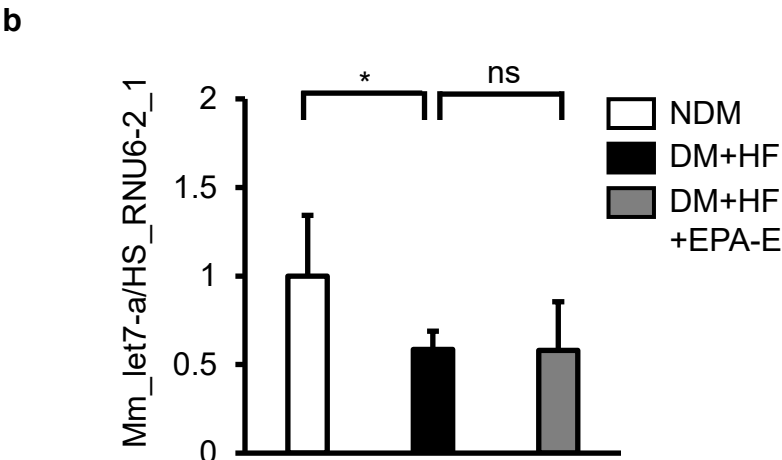

Supplement: Supplementary Materials — Supplementary Figure 1: evaluation of the oxidative stress in the renal cortex. (a)−(c) Characterization of the changes in the mRNA levels of p47phox (a), Nox2 (b), and Nox4 (c) expression in the renal cortex of the nondiabetic mice, STZ-induced diabetic mice, and STZ-induced diabetic mice treated with EPA-E. ∗P < 0.05. ns: not significant. These data are expressed as mean ± SD. NDM: nondiabetic mice; DM + HF: mice with STZ-induced diabetes were fed a high-fat diet; DM + HF + EPA − E: STZ-induced diabetic mice were fed a high-fat diet treated with EPA-E. Supplementary Figure 2: high glucose promotes End-MT and EPA-E inhibits its effect. End-MT was evaluated using the Boyden chamber assay. The endothelial cells were passaged in upper chamber. Twenty-four hours after passage, the medium was changed to low glucose (5.6 mM glucose and 19.4 mM mannitol) or high glucose medium (25 mM glucose) with or without of 50 μM EPA-E. The migration cells were stained with DAPI and counted. ∗P < 0.05. ∗∗P < 0.01. These data are expressed as mean ± SD. Supplementary Figure 3: effect of selective inhibitor of TGF-βR1, LY36497 on CD31 expression in endothelial cells. Immunoblot analysis of CD3l. Endothelial cells were incubated in the adipocyte conditioned medium 1 µM or 10 µM LY36497. ∗P < 0.05. ns: not significant. These data are expressed as mean ± SD. Regarding immunoblot, membranes are cut prior to hybridization with antibodies, so these are not images of full-length blots. Supplementary Figure 4: Mm_miR-29b and Mm_let-7a reveal trends of suppression in the renal cortex. (a) and (b) miRNAs alteration in the renal cortex of the renal cortex of the nondiabetic mice, STZ-induced diabetic mice, and STZ-induced diabetic mice treated with EPA-E. (a) Mm_miR-29b. (b) Mm_let-7a. ∗P < 0.05. ns: not significant. These data are expressed as mean ± SD. NDM: nondiabetic mice; DM + HF: mice with STZ-induced diabetes were fed a high-fat diet; DM + HF + EPA − E: STZ-induced diabetic mice were fed a [file 2182225.f1.zip › 2182225.f1/supple 4 6.16.2021.pdf]
